# Determinants of Doppler-based renal resistive index in patients with septic shock: impact of hemodynamic parameters, acute kidney injury and predisposing factors

**DOI:** 10.1186/s13613-019-0525-8

**Published:** 2019-04-24

**Authors:** François Beloncle, Natacha Rousseau, Jean-François Hamel, Alexis Donzeau, Anne-Lise Foucher, Marc-Antoine Custaud, Pierre Asfar, René Robert, Nicolas Lerolle

**Affiliations:** 1 Département de Médecine Intensive - Réanimation et de Médecine Hyperbare, Centre Hospitalo- Universitaire d’Angers, Université d’Angers, 4 rue Larrey, 49933 Angers Cedex 9, France; 2 Service de Réanimation médicale, Centre Hospitalo-Universitaire de Poitiers, Université de Poitiers, Poitiers, France; 3 Unité de Méthodologie and Biostatistique, DRCI, Centre Hospitalo-Universitaire d’Angers, Université d’Angers, Angers, France; 4 Centre de recherche clinique, Centre Hospitalo-Universitaire d’Angers, Université d’Angers, Angers, France

**Keywords:** Acute kidney injury, Shock, Sepsis, Resistive index, Critical care, Vasopressors, Clinical study

## Abstract

**Background:**

Increased renal resistive index (RI) measured by Doppler ultrasonography has been shown to be associated with acute kidney injury (AKI) in septic patients. However, its clinical use is limited by poor sensitivity and specificity which may be explained by its numerous determinants [in particular mean arterial pressure (MAP)]. We measured, in patients with septic shock, RI at different MAP levels over a short period of time on the admission day to ICU (D1) and every 3 days until day 10 (D10) to define the determinants of RI and study specifically the relationship between RI and MAP.

**Results:**

Consecutive patients with septic shock without preexisting chronic renal dysfunction were included in this prospective cohort study in two ICUs. Sixty-five patients were included in the study. Thirty-three (50.8%) and 15 (23.1%) patients had a history of chronic hypertension or diabetes, respectively. At D3, 35 patients presented AKI with AKIN 2 or 3 criteria (severe AKI, AKIN2-3 group) and 30 presented no AKIN or AKIN 1 criteria (AKIN0-1 group). As previously described, RI at D1 was higher in the AKIN2-3 group than in the AKIN0-1 group (0.73 interquartile range [0.67; 0.78] vs. 0.67 [0.59; 0.72], *p* = 0.001). A linear mixed model for predicting RI from D1 to D10 showed that an increase in pulse pressure, presence of severe AKI and additional day of ICU hospitalization were associated with an increase in RI. An increase in MAP and recovery from severe AKI were associated with a decrease in RI. In the presence of chronic hypertension or diabetes, an increase in MAP resulted in a lower decrease in RI, than in the absence of such factors. Presence of AKI at D3 did not impact the relationship between MAP and RI.

**Conclusions:**

Severe AKI was associated with a reversible increase in RI without significant interaction with the relationship between MAP and RI. Conversely, the presence of chronic hypertension and/or diabetes interacted with this relationship.

## Background

Acute kidney injury (AKI) occurs in approximately 25% of sepsis in intensive care unit (ICU) [1, 2] and 50% of septic shock [2]. Septic AKI pathophysiology is complex: interactions between renal hemodynamic changes and inflammation are probably involved in both initial and established phases of AKI [3]. Overall, vascular dysfunction may play a pivotal role in these pathophysiological mechanisms [4–6]. Thus, renal resistive index (RI) measured by Doppler ultrasonography allowing to explore noninvasively renal hemodynamics has been used to predict renal dysfunction [7–9]. Increased RI has been shown to be associated with renal failure, especially in septic patients [7]. However, RI’s clinical use is limited by poor sensitivity and specificity. A wide overlap of RI values between patients with and without renal failure is thus observed. This may be explained by the numerous determinants that contribute to the RI [10]. RI may be determined not only by “direct” renal determinants such as renal vascular lesions impacting arteriolar resistance and compliance [11] but also by non-renal factors indirectly modifying renal hemodynamics such as mean arterial pressure (MAP) [7, 12], pulse pressure (PP) [13], heart rate (HR) [14], fluid challenge [15] or arterial partial pressures of oxygen (PaO_2_) [16, 17] and carbon dioxide (PaCO_2_) [17].

The relationship between MAP and RI has been demonstrated to be particularly strong and was observed repeatedly over several studies [7, 12, 18]. RI was shown to be inversely correlated with MAP, which might be consistent with physiologic renal post-glomerular vasoconstriction observed during low renal perfusion in order to maintain glomerular filtration pressure [7]. Interestingly, Dewitte et al. [12] observed that this relationship was abolished in patients with AKI. Together with animal studies showing that renal vascular lesions are at stake very early in the course of AKI [6, 19], this observation may indicate that renal vascular reactivity to MAP changes is impaired in AKI. Alternatively, vascular factors predisposing to AKI, such as diabetes and chronic arterial hypertension, may be responsible for this altered vascular response before the onset of AKI lesions [20, 21].

In an attempt to define the role of these determinants on RI in patients with septic shock, we performed RI measurements at different MAP levels over a short period of time during vasopressor syringe pump relays performed during routine care on the admission day in ICU (D1) and every 3 days until day 10 (D10).

This study aimed at exploring the determinants of RI in patients with septic shock and at studying specifically the relationship between MAP and RI in patients with and without AKI. Specifically, we put forward the hypothesis that RI changes to MAP variation were modified in patients with AKI and/or in the presence of diabetes or hypertension.

## Methods

### Patient selection

Patients admitted between May 2012 and May 2013 in the medical ICU of the university hospitals of Angers and Poitiers in France (24 and 15 ICU beds, respectively) for septic shock, defined by the criteria of the Society for Critical Care Medicine/American College of Chest Physicians [22], were prospectively included within 24 h of ICU admission, after hemodynamic stability had been obtained (i.e., when MAP was higher than 65 mmHg for more than 1 h with a stable catecholamine dose, and after adequate vascular filling as judged by the attending physician). Norepinephrine was administered as a first-line vasopressor as recommended by the French intensive care societies [23].

The exclusion criteria were age less than 18 years, preexisting chronic renal dysfunction (defined as a pre-ICU admission baseline serum creatinine (SCr) > 130 μmol/l), or known renal artery stenosis.

### Resistive index (RI) measurements, study protocol and data collections

#### RI measurements

Bedside Doppler ultrasonography was performed with a 7.5 MHz transducer (Compact Xtreme CX50, Philips Medical Systems, Bothell, WA, USA), using a previously described RI measurements technique [7, 24]. Briefly, sonography and color Doppler mode were used to localize one of the kidneys and interlobar arteries. Pulse-wave Doppler was used to measure blood velocities in the interlobar arteries. Peak systolic and end-diastolic velocities were measured on 5 consecutive pulses. RI was calculated as follows: RI = (peak systolic velocity−end-diastolic velocity)/peak systolic velocity. The RI values presented here correspond to the average of the 5 measures performed on the 5 consecutive pulses. All RI measurements were performed by intensivists experienced in kidney Doppler ultrasonography and certified in echocardiography. Inter-observer reproducibility testing displayed good agreement between operators (intraclass correlation coefficients and their 95% confidence interval 0. 97 (0.88–0.99)).

#### Study protocol for sequence of RI measurements

**Fig. 1:**
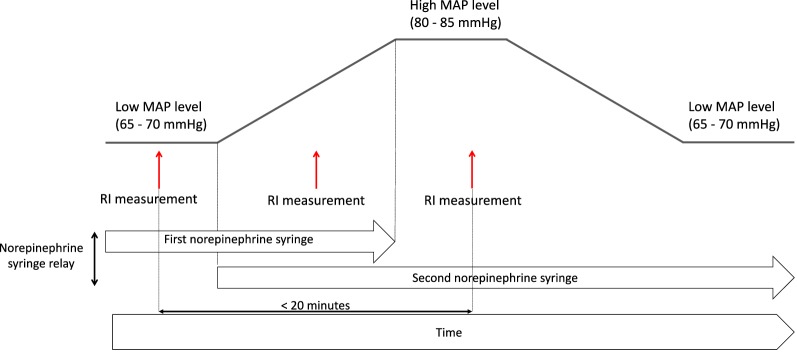
Renal resistive index (RI) measurements and sequence of RI measurements. A RI measurement corresponded to the mean of five measures performed on five consecutive pulses at one mean arterial pressure (MAP) level. A sequence of RI measurements included from 2 to 5 RI measurements performed in less than 20 min at, at least, 2 MAP levels (aim of at least 15 mmHg apart, between the two levels). When vasopressors were discontinued, the sequence of RI measurements included only one RI measurement at one MAP level

A sequence of RI measurements included from 2 to 5 RI measurements performed in less than 20 min at, at least, 2 MAP levels (aim of at least 15 mmHg apart, between the 2 levels) during routine vasopressor syringe pump relays (Fig. 1). In our ICUs, vasopressor syringe pump relays were performed by starting a second vasopressor syringe pump before the end of the ongoing syringe. These relays are typically associated with transient MAP increase during the overlapping delivery of norepinephrine by the two pump syringes. A first RI measurement was performed at the beginning of vasopressor syringe pump relay, and one or more additional RI measurements were performed during the relay when MAP levels varied.

A sequence of measurements was performed at the time of inclusion of patient in the study (D1) and repeated 3 times per week until D10 (or discharge from ICU). When vasopressors were discontinued, only one RI measurement at one MAP level was performed.

#### Parameters collected or calculated at each RI measurement

On each RI measurement, the following data were recorded: HR, systolic, diastolic and mean arterial pressures, norepinephrine dose, AKIN score based on SCr and urine output [25] and the day of ICU hospitalization on the day of RI measurement (with D1 corresponding to the admission day to ICU).

In patients with severe AKI defined as AKI AKIN 2 or 3 on D3, “recovery of severe AKI” or “no recovery” was noted for each RI measured thereafter. This item was evaluated post hoc according to urine output and SCr by an adjudication committee (N.L and P.A) blinded to RI values.

##### Other data

The following data were recorded: age, past medical history of diabetes mellitus and chronic hypertension, treatment at admission, baseline SCr (lowest value in the 3 months preceding admission, if no SCr was available, the baseline value was estimated using the MDRD formula, assuming a creatinine clearance of 75 ml/min/1.73 m^2^), infection site, nosocomial or community-acquired infection, responsible microorganism(s), ICU admission weight, arterial lactate concentration, Sequential Organ Failure Assessment (SOFA) score and Simplified Acute Physiologic Score II (SAPS II) at D1 [26, 27].

### Statistical analysis

Data are presented as median [interquartile range] or number (percentage). The study population was divided into 2 groups according to the AKIN classification at D3. For the patients who died before D3, the AKIN score on the date of expiry was used. The *AKIN2*-*3* group was composed of patients with severe AKI (AKIN 2 or 3) at D3, and the patients without AKIN criteria or with AKIN 1 at D3 made up the *AKIN0*-*1* group. The 2 groups of patients were compared using Mann–Whitney *U* test or Fisher’s exact test as appropriate for variables at inclusion. RI values at D1 were compared according to AKIN classification at D3 using Kruskal–Wallis test.

We studied the impact on RI of MAP, PP, severe AKI, recovery of severe AKI (for RI measured after D3), time since ICU admission on the day of RI measurement, past medical history of diabetes and/or hypertension. Interactions between MAP and chronic arterial hypertension and/or diabetes were also considered. The selection of covariates explaining RI was predefined and based on clinical knowledge (and not on statistical selection procedure, as recommended for limiting the risk of type I error, or false positive rate). Different measurements carried out on a same patient are necessarily correlated. As standard statistical models (such as linear models) are based on the observations’ independence assumption, their use was deemed inappropriate. We thus used a multivariate linear mixed model [28] for modeling RI, allowing to estimate the impact of all the predefined covariates considered as fixed effects using a single model, taking into account the correlation of the different measurements performed in each patient, and modeling residuals as random effects. Random effects describe the difference between actual values of the studied parameters as measured during the study and theoretical values predicted by the model when entering the fixed effects. The model was constructed by minimizing Akaïke criteria. The model presented was validated by verifying the normality and homoscedasticity assumptions of the residuals. The significance of covariates was tested by the use of Wald tests.

All tests were performed with a type I error set at 0.05, except for the interactions between variables in linear mixed models for which a threshold of 0.1 is generally accepted [29–31]. Analyses were performed with Stata 11.0 (StataCorp LP, TX, USA).

## Results

### Study population, groups *AKIN0*-*1 and AKIN2*-*3* at day 3

Sixty-five patients were included in the study and had a sequence of RI measurements at D1. In total, 139 RI measurements were performed during these 65 sequences at D1. Fifty-two sequences of RI measurements were performed at D3, 43 at D5–D6 and 32 at D9–D10 (78, 63 and 40 RI measurements, respectively). None of the RI measurement sequence lasted more than 20 min. On D3, 26 (40%) patients did not meet AKIN criteria, 4 (6.2%) met AKIN 1 criteria, 12 (18.4%) met AKIN 2 criteria and 23 (35.4%) met AKIN 3 criteria. No patients received diuretics between inclusion and D3; thus, their AKIN classification was not influenced by this therapy. Seven (10.8%) patients died before D3. All of them met AKIN 3 criteria on the day of expiry. Thirty-five patients constituted the *AKIN2*-*3* group and 30 the *AKIN0*-*1* group. Of note, no patient in the *AKIN0*-*1* group developed severe AKI after D3. Baseline patient characteristics at inclusion, categorized according to the AKIN group at D3, are presented in Table 1.

**Table 1:** Patient characteristics at inclusion according to the presence or absence of severe acute kidney injury (AKI AKIN 2 or 3 at day 3)

Parameters	Total (*n* = 65)	Severe AKI at D3 (*AKIN2*-*3 group*) (*n* = 35)	Absence of severe AKI at D3 (*AKIN0*-*1 group*) (*n* = 30)	*p*
Age (years)	66 [54.5; 76.5]	73 [59; 75]	57.5 [47; 77.8]	0.529
Sex. M/F	40/25	24/11	16/14	0.307
*Preexisting conditions*				
Chronic arterial hypertension	33 (50.8%)	24 (68.6%)	9 (30%)	0.003
Diabetes	15 (23.1%)	12 (34.3%)	3 (10%)	0.021
Liver cirrhosis	5 (7.7%)	4 (11.4%)	1 (3.3%)	0.363
*Treatment at admission*				
ACE inhibitor	20 (30.8%)	14 (40%)	6 (20%)	0.108
NSAIDs	6 (9.2%)	5 (14.3%)	1 (3.3%)	0.205
Mechanical ventilation	52 (80%)	31 (88.6%)	21 (70%)	0.118
SAPS II	53 [41.5; 66.5]	56 [48; 70]	50.5 [39.3; 64.3]	0.108
SOFA score total (24 h)	10 [8, 13]	12 [10, 14]	9 [6, 12]	0.004
Respiratory	3 [2, 4]	3 [2, 4]	3 [1.8; 3]	0.02
Cardiovascular	4 [4, 4]	4 [4, 4]	4 [4, 4]	0.128
Neurologic	0 [0; 0]	0 [0; 0]	0 [0; 0.3]	0.900
Hepatic	0 [0; 1.5]	1 [0; 2]	0 [0; 1.25]	0.521
Hematologic	1 [0; 2]	1 [0; 2]	1 [0; 2]	0.756
Renal	1 [0; 4]	3 [0; 4]	0.5 [0; 1]	0.002
Serum creatinine (μmol/L)	119.5 [75.5; 158.2]	127.5 [99.5; 210.3]	105 [62; 127.3]	0.009
Arterial lactate (mmol/L)	2 [1.3; 3.3]	2 [1.4; 4.9]	1.8 [1.1; 2.7]	0.035
Community-acquired infection	43 (66%)	24 (69%)	19 (63%)	0.794
*Source of infection*				
Lung	22 (33.8%)	10 (28.6%)	12 (40%)	–
Urinary tract	11 (16.9%)	7 (20%)	4 (13.3%)	–
Abdomen	11 (16.9%)	9 (25.7%)	2 (6.7%)	–
Central nervous system	1 (1.5%)	0 (0%)	1 (3.3%)	–
Gynecologic tract	1 (1.5%)	0 (0%)	1 (3.3%)	–
Bone	1 (1.5%)	1 (2.9%)	0 (0%)	–
ENT	2 (3.1%)	0 (0%)	2 (6.7%)	–
Vascular catheter	2 (3.1%)	0 (0%)	2 (6.7%)	–
Skin	5 (7.7%)	1 (2.9%)	4 (13.3%)	–
Unknown	10 (15.4%)	6 (17.1%)	4 (13.3%)	–
*Type of organism*				
Gram-positive	16 (24.6%)	9 (25.7%)	7 (23.3%)	*
Gram-negative	27 (41.5%)	16 (45.7%)	11 (36.7%)	*
Other	3 (4.6%)	1 (2.9%)	2 (6.7%)	*
Not identified	21 (32.3%)	9 (25.7%)	12 (40%)	*

Values are number (percentage) or median [interquartile range]

ACE inhibitor, angiotensin-converting enzyme inhibitor; ARB, angiotensin II receptor blockers; NSAIDs, nonsteroidal anti-inflammatory drugs; SAPS II, Simplified Acute Physiology Score II; SOFA, Sepsis-Related Organ Failure Assessment

*p* values refer to the comparison between the *AKIN2*-*3* at D3 and *AKIN0*-*1* at D3 groups. *Overall comparison *p* = 0.6

Thus, in this population of patients with septic shock, a high prevalence of AKI was observed. The presence of AKI was statistically associated with conditions related to renal susceptibility to injury and a high disease severity at admission.

### Relationship between renal resistive index at day 1 and acute kidney injury at day 3

**Fig. 2:**
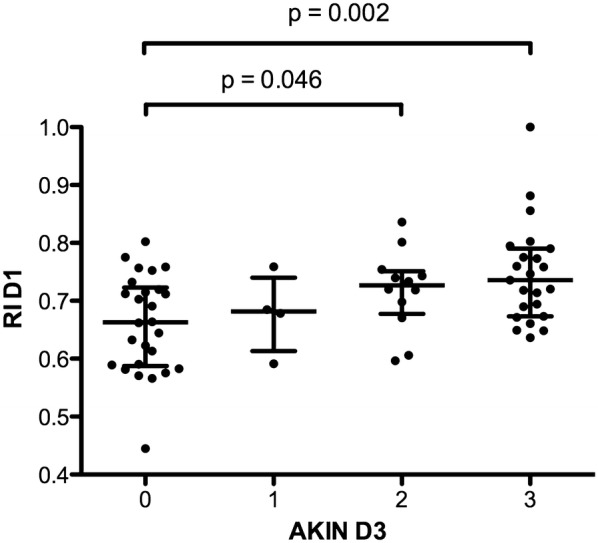
Distribution of renal resistive index at day 1 (RI D1) according to AKIN classification at day 3 (AKIN D3). RI D1 corresponds to the mean of RI measured for each patient at different mean arterial pressure levels over a period of 20 min on the ICU admission day. Horizontal lines represent median, first and third quartile values for each group. Overall comparison: *p* = 0.01

RI D1 corresponding to the mean of the RI measured during the sequence of RI measurements at inclusion (i.e., the mean of RI measured at different MAP levels at D1) was higher in the *AKIN2*-*3* group at D3 than in the *AKIN0*-*1* group (0.73 interquartile range [0.67; 0.78] versus 0.67 [0.59; 0.72], *p* = 0.001). RI D1 was significantly different among the patients without AKI or with AKIN 1, 2 and 3 at D3 (Fig. 2). Similar results were found with RI measured at the lowest or highest MAP level of the sequence of RI measurements (data not shown).

These data confirmed that RI at admission was greater in patients with severe AKI at D3 than in patients without AKI or with AKI AKIN 1 at D3, but with a large overlap in RI values, preventing its clinical use, as previously observed [7–9].

### Determinants of renal resistive index: linear mixed model for renal resistive index

For each patient, each sequence of RI measurements consisted in 1–5 RI measurements. All measurements were entered in the linear mixed model, and the following parameters were tested for association with RI: presence/absence of severe AKI at D3, MAP, PP, time since ICU admission on the day of RI measurement, recovery of severe AKI (for RI measured after D3) and past medical history of diabetes and/or hypertension (Table 2). The model highlighted a significant association between RI and MAP, PP, severe AKI, severe AKI recovery, diabetes and/or hypertension history, and day of ICU hospitalization. To sum up, a PP increase, any additional day in ICU and presence of severe AKI at D3 (vs. no AKI or AKI AKIN 1 at D3) were associated with a RI increase. A MAP increase and severe AKI recovery were associated with a RI decrease. A MAP increase led to a weaker RI decrease in patients with chronic hypertension or diabetes mellitus history than in patients without such a history. Conversely, the presence of severe AKI at D3 did not impact the relationship between MAP and RI, i.e., the slope of the relationship between RI and MAP was unchanged by severe AKI vs. AKIN 0 or 1. Random effects, i.e., the part of RI not explained by the tested parameters, were high in comparison with the effect of tested parameters.

**Table 2:** Linear mixed model for predicting resistive index (RI) measured from day 1 (D1) to day 10 (D10)

RI from D1 to D10	Coefficient	95% CI	*p*
*Fixed effects*			
MAP (+ 1 mmHg) if no chronic arterial hypertension and no diabetes	− 0.002	− 0.002; − 0.001	< 0.001
MAP (+ 1 mmHg) if chronic arterial hypertension and/or diabetes	− 0.001	− 0.002; − 0.001	< 0.001*
PP (+ 1 mmHg)	0.002	0.001; 0.002	< 0.001
Severe AKI at D3	0.032	0.006; 0.059	0.017
Recovery of severe AKI at day of RI measurement	− 0.030	− 0.057; − 0.003	0.031
Time (+ 1 day of ICU hospitalization)	0.003	0.001; 0.005	0.002
Constant	0.699	0.666; 0.733	< 0.001
Random effects	0.046	0.043; 0.050	

All the continuous covariates, the coefficient associated with the considered covariate corresponds to the impact on renal resistive index of the increase of one unit in this covariate

*MAP* mean arterial pressure, *PP* pulse pressure, *AKI* acute kidney injury, *ICU* intensive care unit, *Severe AKI* acute kidney injury acute kidney injury network 2 or 3

*Interaction between MAP and chronic arterial hypertension and/or diabetes, *p* = 0.06

## Discussion

In this study on 65 patients with septic shock, analysis of RI determinants was performed using a linear mixed model. On a pathophysiological point of view, this model brings up several important observations. We first confirmed the association between high RI on D1 and subsequent AKI [7, 9] and the negative correlation between MAP and RI [7, 18]. We further extended these data by showing conversely that PP is positively correlated with RI. More importantly, the model describes how AKI and predisposing factors to AKI impact renal hemodynamic. We observed that AKI is associated with an increase in RI but did not interact with the relationship between MAP and RI. On the opposite, chronic arterial hypertension and diabetes did not modify directly RI but interacted with the MAP/RI relationship, a less steeper slope being observed when these risk factors are present. In synthesis and regarding our initial working hypothesis, the presence of predisposing factors to AKI was associated with lower RI reactivity to MAP changes, and AKI was associated with higher RI.

Hypertension and diabetes are well-known factors for AKI, in particular through induction of chronic vascular renal disease [33]. It may be hypothesized that low RI reactivity to MAP changes indicates the presence of chronic renal vascular lesions. It would have been interesting to assess vascular disease in our patients in other vascular beds by validated techniques (such as Doppler ultrasounds of lower limbs and carotid arteries) to determine if the low reactivity to MAP changes correlates with the extent of chronic vascular damage. Unfortunately, this was not possible in our patients during the ICU stay. Confirming the lower response to MAP changes in patients with diabetes and/or hypertension outside the ICU may be interesting but unfortunately will be difficult as administering norepinephrine to modify MAP is not routine care outside the ICU. Interestingly, the interaction between MAP target and chronic hypertension regarding AKI has been highlighted in a randomized clinical trial on septic shock patients [34]. In this study, a higher MAP target (i.e., 85 mmHg) resulted in a lower risk for AKI, only in the subgroup of patients suffering from chronic hypertension. Together with this observation, our study indicates that chronic renal vascular changes may be implicated in the chain of events leading from hypertension to AKI. In addition, the correlation between PP and RI may also illustrate the relation between predisposing factors to AKI and RI. This positive correlation may be explained by the negative correlation between PP and vascular compliance [32]. In sum, a high PP may reflect a low vascular compliance which is known to be associated with a high RI [11].

We identify that recovery from severe AKI was associated with RI decrease and that the extent of this decrease was very close to the extent of the increase associated with AKI. Thus, the increase in RI associated with AKI seems to be reversible and to evolve in a time course similar to AKI itself. Importantly, this observation indicates that the increase in RI associated with AKI is likely to be related to the pathophysiology of AKI itself and not to predisposing factors. Our study was not designed to disclose whether the decrease in RI may permit to diagnose early AKI healing, i.e., before SCr decrease. This may be an interesting point to study in the future. Finally, we identified time spent in the ICU as a factor associated with higher RI. From our data set, we cannot determine whether this association relates to the progression of renal acute disease over time, effect of ICU treatments or other unknown factors.

Regarding the value of RI by itself, the model showed that despite entering several determinants associated with RI, the random effect, i.e., the variation in RI not “explained” by these determinants, was high (0.046) and even higher than the increase in RI associated with severe AKI (0.032). Thus, even when correcting for these several RI determinants, the variation in RI between patients with and without severe AKI is below the inter-individual variability of the index, which explains the low individual performance observed in most studies. Several additional RI determinants may be hypothesized: renal venous pressure, renal congestion, cardiac output, abdominal pressure, pO_2_, etc. They may narrow the random effect but will result in an over-sophisticated model that is likely to perform poorly should it be used to predict AKI [35]. These data suggest that the use of the RI for clinical practice for AKI prediction is probably unrealistic, even by taking into account several RI determinants or by using a dynamic approach. Indeed, we assessed several ways to combine parameters with RI to improve its accuracy to predict AKI, such as the ratio ∆RI/∆MAP during MAP swings, but none proved effective (data not shown). Furthermore, as RI does not represent only renal vascular resistance, it can also not be used to determine the “optimal” MAP level in every patient.

Our study has several limitations. Firstly, as already highlighted, the high value of the residual random effects suggests that the participation of other determinants of the RI was not taken into account. Secondly, renal hemodynamics changes due to MAP changes may vary according to the initial MAP level, depending on whether these values remain within the auto-regulatory range [18]. The renal autoregulation plateau refers to a range of perfusion pressure values in which the renal blood flow is constant irrespective of the perfusion pressure [36, 37]. Below this plateau, the renal blood flow is directly dependent on the perfusion pressure. This phenomenon should be associated with an increase in vascular resistance, and hence potentially in RI, with increasing MAP above a threshold [37]. This may add another level of sophistication to the relationship between MAP and RI that may be extremely difficult to take into account, as the threshold for this plateau is probably highly variable between patients. Furthermore, MAP may be considered as a surrogate of renal perfusion pressure. However, this approximation does not account for the venous renal pressure. Lastly, kidney inflammation potentially linked to renal hemodynamic changes was not analyzed in our study [38].

## Conclusions

RI was impacted by MAP, presence of severe AKI and severe AKI recovery, PP, day from ICU admission and past medical history of diabetes and/or hypertension. AKI was associated with a reversible increase in RI without significant interaction with the relationship between MAP and RI. Conversely, the presence of chronic hypertension and/or diabetes interacted with this relationship. Finally, variability of RI despite taking into account these numerous parameters remained high, suggesting that its use in clinical practice to predict AKI is probably unrealistic.

## Abbreviations

AKI: Acute kidney injury
AKIN: Acute kidney injury network
ICU: Intensive care unit
HR: Heart rate
MAP: Mean arterial pressure
PaO_2_: Arterial partial pressure of oxygen
PaCO_2_: Arterial partial pressure of carbon dioxide
PP: Pulse pressure
RI: Renal resistive index
SAPS II: Simplified acute physiologic score II
SCr: Serum creatinine
Severe AKI: Acute kidney injury acute kidney injury network 2 or 3
SOFA: Sequential Organ Failure Assessment
